# Changes in Hospital Care for Children With IBD Across Australia From 2014 to 2022

**DOI:** 10.1111/jpc.70424

**Published:** 2026-06-01

**Authors:** Laura Rishanghan, David Skvarc, Stefan Moller, Ramesh Nataraja, Scott Nightingale, Christopher Burgess, Shoma Dutt, George Alex, Helen Jurgens, Ajay Sharma, Paul Hammond, Cheryl Foo, Leanne Raven, Antonina Mikocka‐Walus, Wayne Massuger, Edward Giles

**Affiliations:** ^1^ Department of Gastroenterology Monash Children's Hospital, Monash Health Melbourne Victoria Australia; ^2^ School of Psychology, Faculty of Health Deakin University Geelong Victoria Australia; ^3^ Independent Researcher Melbourne Australia; ^4^ Department of Paediatrics and Surgery School of Clinical Sciences, Faculty of Medicine, Nursing and Health Sciences, Monash University Melbourne Australia; ^5^ Department of Paediatric Surgery Monash Children's Hospital Melbourne Australia; ^6^ John Hunter Children's Hospital, Paediatric Gastroenterology Newcastle Australia; ^7^ Queensland Children's Hospital Department of Gastroenterology, Hepatology and Liver Transplant Brisbane Australia; ^8^ The University of Queensland, Faculty of Medicine Brisbane Australia; ^9^ Children's Hospital Westmead Department of Gastroenterology Sydney Australia; ^10^ Children's Hospital Westmead Clinical School University of Sydney Sydney Australia; ^11^ Department of Gastroenterology and Clinical Nutrition The Royal Children's Hospital Melbourne Australia; ^12^ Department of Paediatrics The University of Melbourne Melbourne Australia; ^13^ Women's & Children's Hospital, Paediatric Gastroenterology Adelaide Australia; ^14^ Fiona Stanley Hospital, Paediatric Gastroenterology Perth Australia; ^15^ Joondalup Health Campus Perth Australia; ^16^ Crohn's & Colitis Australia Melbourne Australia; ^17^ School of Psychology, Deakin University Geelong Australia; ^18^ Centre for Innate Immunity and Infectious Disease, Hudson Institute for Medical Research Melbourne Victoria Australia; ^19^ Department of Paediatrics Monash University Melbourne Victoria Australia

**Keywords:** admission, hospital, national standards, paediatric

## Abstract

**Background:**

Inflammatory bowel disease is a chronic, lifelong gastrointestinal disorder, with 8%–10% of patients diagnosed < 18 years old. Over the last decade, there has been a global increase in paediatric IBD (pIBD) incidence and changes in therapeutic approaches. Our study aimed to assess the changes in hospital care for young people with IBD from 2014 to 2022.

**Methods:**

Complete national pIBD data on admissions was collected through the Australian Institute of Health and Welfare (AIHW). Hospitals nationally were invited to participate in a clinical audit of inpatient overnight admissions for 2021. Data was compared to a previous study from 2014.

**Results:**

There were 813 overnight pIBD admissions in 2021, compared to 590 in 2014. Eight public hospitals participated, with 186 admissions, capturing 23% of all 2021 pIBD admissions. UC admission rates doubled; however, surgical admissions reduced (16% in 2014 to 5% in 2021, *p* < 0.05). CD and surgical admissions remained stable. There was reduced corticosteroid use, and 56 patients had off‐label medication use. Sixty‐five percent of patients had active disease at the last clinic review. There was a trend towards shorter admissions, and re‐admission rates were similar to previous global data. Despite psychological co‐morbidity in 28% of cases, psychologists were not part of the team at any site.

**Conclusion:**

This study showed a significant increase in UC admissions in Australia over the last decade. Medication changes were in line with global trends, and multi‐disciplinary care remained inadequate despite national standards. This data provides evidence for planning and resourcing pIBD care nationally and worldwide.

Inflammatory bowel disease (IBD) is a chronic and lifelong condition caused by a dysregulated immune response to intestinal microbiota in genetically susceptible individuals and influenced by environmental triggers leading to chronic inflammation of the gastrointestinal tract. Paediatric onset IBD (diagnosed < 18 years age) accounts for 7%–10% of all incident cases and is associated with a more severe disease course than adult‐onset IBD [1]. Internationally, in the last 20–25 years, there has been a significant global increase in the incidence and prevalence of paediatric‐onset IBD [2]. A diagnosis of IBD in childhood can have a significant impact on the quality of life for patients and their families, as well as the considerable social and economic burden on healthcare systems [3]. Pharmacological therapy for paediatric IBD continues to evolve; however, access often remains limited compared to adult patients [4]. Anti‐tumour necrosis factor alpha (anti‐TNF) is the only advanced therapy available for use in children in Australia. While anti‐TNF's have revolutionised treatment since the 2000s, children often need escalated dosing compared to the adult population [5]. One third of paediatric IBD patients are also either primary non‐responders or have secondary loss of response to anti‐TNFs [5]. The rise in the use of biologic therapy (anti‐TNFs) has meant a reduction in the use of corticosteroids since the 2000s, coincident with greater awareness and understanding of the risks of long‐term steroid therapy [6, 7, 8].

The increasing use of earlier advanced therapies and reduced reliance on steroids aim to improve the quality of life for children/young people with IBD. However, studies demonstrating improved outcomes are relatively sparse. Two studies from the United States, from 2013 to 2024 show 22% and 15% readmission rates (in 90 and 30 days respectively) to hospital for paediatric IBD patients [9, 10], suggesting that, at least on some measures, there is an ongoing need for improvement in hospital care.

Crohn's and Colitis Australia, a national patient advocacy group, established a national standard of care for IBD modelled after a UK IBD audit conducted over a 10 year period [11]. A 2014 Australian national audit including adult and paediatric hospitals, showed that only one hospital in Australia met the specified standards of care [12]. The federal government funded a follow‐up project in 2021 to assess the changes in IBD care for children in Australia. The aims of this national study were therefore to analyse the change in resources and delivery of hospital care for young people with IBD in Australia over the last decade.

## 1 Methods

This IBD quality of care project was a follow‐up from the previous project undertaken in 2014 [12]. There were 241 public and private hospitals in Australia that were invited to contribute, with the criteria that they admitted children < 18 years of age. Eight public hospitals participated nationally which covered five states and territories and included seven (of eight) public tertiary centres for paediatric gastroenterology. One hospital was located in an inner‐regional site, and all others were designated as major city localities. Only inpatient episodes at participating hospitals were audited.

There were two main components of the assessment of hospital care:

1. Organisational Survey—a one‐time survey of organisation‐level activities and resourcing completed via webtool.
2. Clinical audit of the 2021 calendar year—health record audit of a series of overnight patient admissions for Crohn's disease and ulcerative colitis (including IBD unspecified) identified via relevant ICD‐10 AM codes.

This report covers the data from the clinical audit. Each hospital was asked to provide data on at least 15 consecutive inpatient admissions for both CD and UC, or their total number of admissions, whichever was higher. Hospitals were able to provide data on more than 15 patients, but this was considered a reasonable number to assess the hospital's practice and was the same as the approach in the 2014 national project. A detailed breakdown of the data is available in the Supporting Information: Clinical Audit Questionnaire. Means for patient‐level data are compared using *t*‐tests; chi‐square is used for proportion comparisons. All analyses were performed in SPSS version 29 (IBM 2022).

An additional dataset of national hospital admission and surgical procedure characteristics was provided by the Australian Institute of Health and Welfare (AIHW) National Hospital Morbidity database, 2020–21, and National Hospital Morbidity database, 2021–22. Ethical approval was granted by the Monash Human Research Ethics Committee under the National Mutual Acceptance framework (HREC: RES‐22‐0000‐050 L). Site‐specific authorisations were obtained from participating sites' respective research governance bodies. Project oversight was administered by the Paediatric IBD Quality of Care Project Advisory Committee (PAC) comprised of key provider and consumer stakeholders. The PAC was overseen by CCA's Scientific, Medical and Quality of Care Advisory Committee that reports to the Board of CCA.

## 2 Results

**FIGURE 1:**
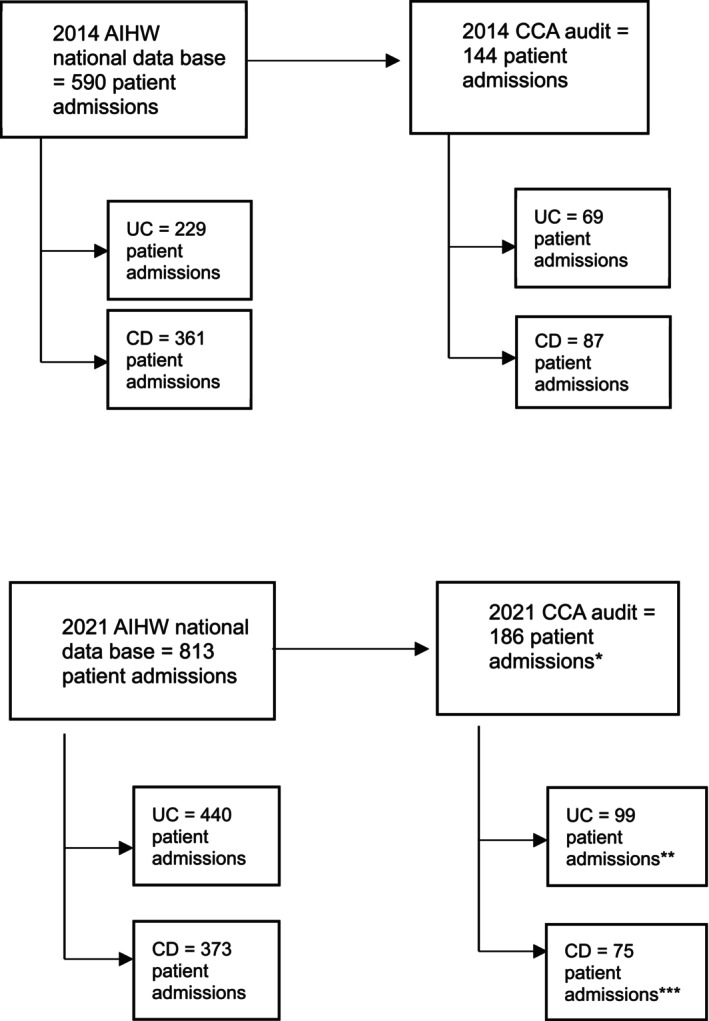
National separations and clinical audit sample proportions. % calculated based on AIHW national data numbers. *186/813 patients = 23% of total patient admissions in 2021 AIHW data, *p* = ns compared to 2014 data ** 99 out of 440 patients = 23% of total UC patient admissions in 2021 AIHW data, *p* = 0.03 compared to 2014 data *** 75 out of 373 patients = 21% of total CD patient admissions in 2021 AIHW data, *p* = ns compared to 2014 data.

Comprehensive national data captured all 813 IBD admissions in Australia from January 1, 2021, to December 31, 2021 (Figure 1). There were 373 CD overnight admissions in 2021, compared to 361 in 2014. For UC, there was almost a doubling of overnight admissions nationally from 229 in 2014 to 440 in 2021.

As expected, most patients presented in major cities (87% or 706/813). Regional sites accounted for 11% (or 86/813) admissions, and outer regional sites for 3% (or 21/813).

IBD‐related surgery requiring overnight admission in hospital occurred on 26 occasions. Data on surgery was not available for 2014. Most patients presented as emergency presentations, 68% (or 537/792). This denominator is slightly lower than the total as some urgency statuses were not assigned or were unknown.

**FIGURE 2:**
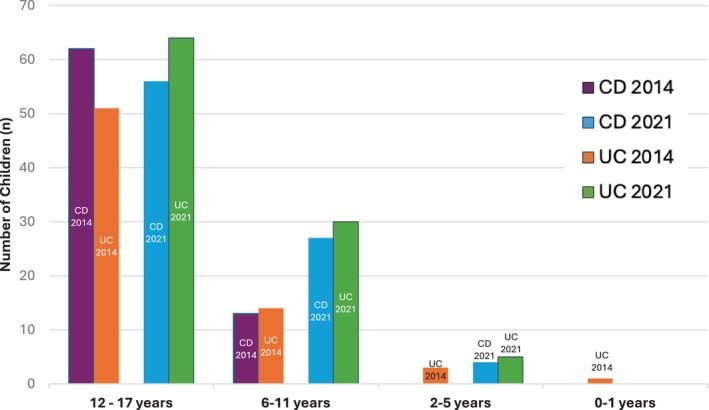
Number of children in different age groups at admission in the CCA 2014 audit versus the 2021 audit. *p* = ns for all data values. 2014 audit = 144 patients in total 2021 audit = 186 patients in total *Patients admitted in the 2–5 year age group for UC in 2014 = 3 Patients admitted in the 2–5 year age group for CD in 2021 = 4 Patients admitted in the 2–5 year age group for UC in the 2021 = 5 Patients admitted in the 0–1 year age group for UC in 2014 = 1.

There were 186 inpatient episodes where clinical care was audited in 2021 (CD in 87/186 or 47%, UC in 99/186 or 53%, male sex in 96/186 or 52%), which is 23% of the total national admissions (23% of CD admissions, and 23% of UC admissions) (Figure 2). Public hospitals provided 91% of overnight admissions.

As shown in the national data, this audit covered a similar number of CD overnight admissions from 2014 to 2021 (from 75 to 87, respectively), with a 43% increase in UC overnight admission in 2021 (from 69 to 99, respectively).

**FIGURE 3:**
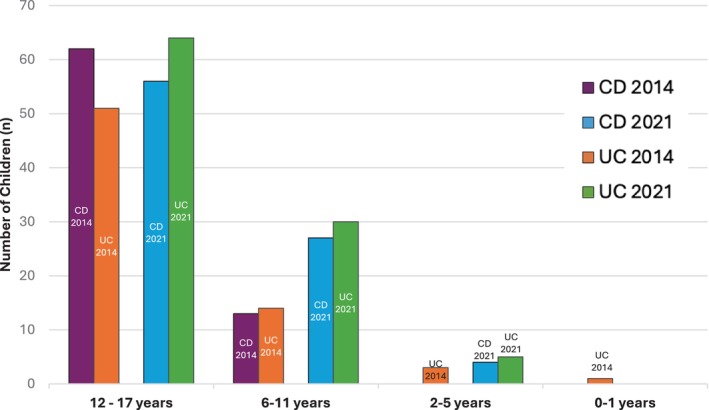
Number of children in different age groups at admission in the 2014 audit versus the 2021 audit. *p* = ns for all data values. 2014 audit = 144 patients in total, 2021 audit = 186 patients in total. Crohn's Disease 2014 Crohn's Disease 2021.

There was an increase in the proportion of CD admissions in children younger than 12, with a statistically significant reduction in children aged 12–17 years presenting, from 62 out of 75 or 83% in 2014 to 56 out of 87 or 64% in 2021 (*p* < 0.01). There was no statistically significant difference between ages on admission for UC (see Figure 3).

**FIGURE 4:**
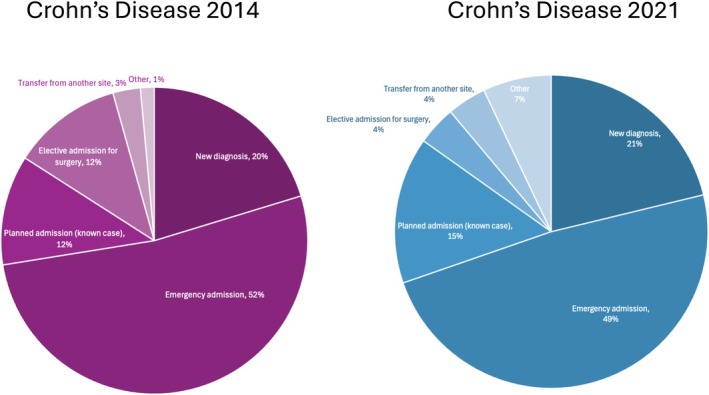
Primary reason for Crohn's disease admissions, expressed as % of total CD admissions in CCA audit. Ulcerative Colitis 2014 Ulcerative Colitis 2021.

**FIGURE 5:**
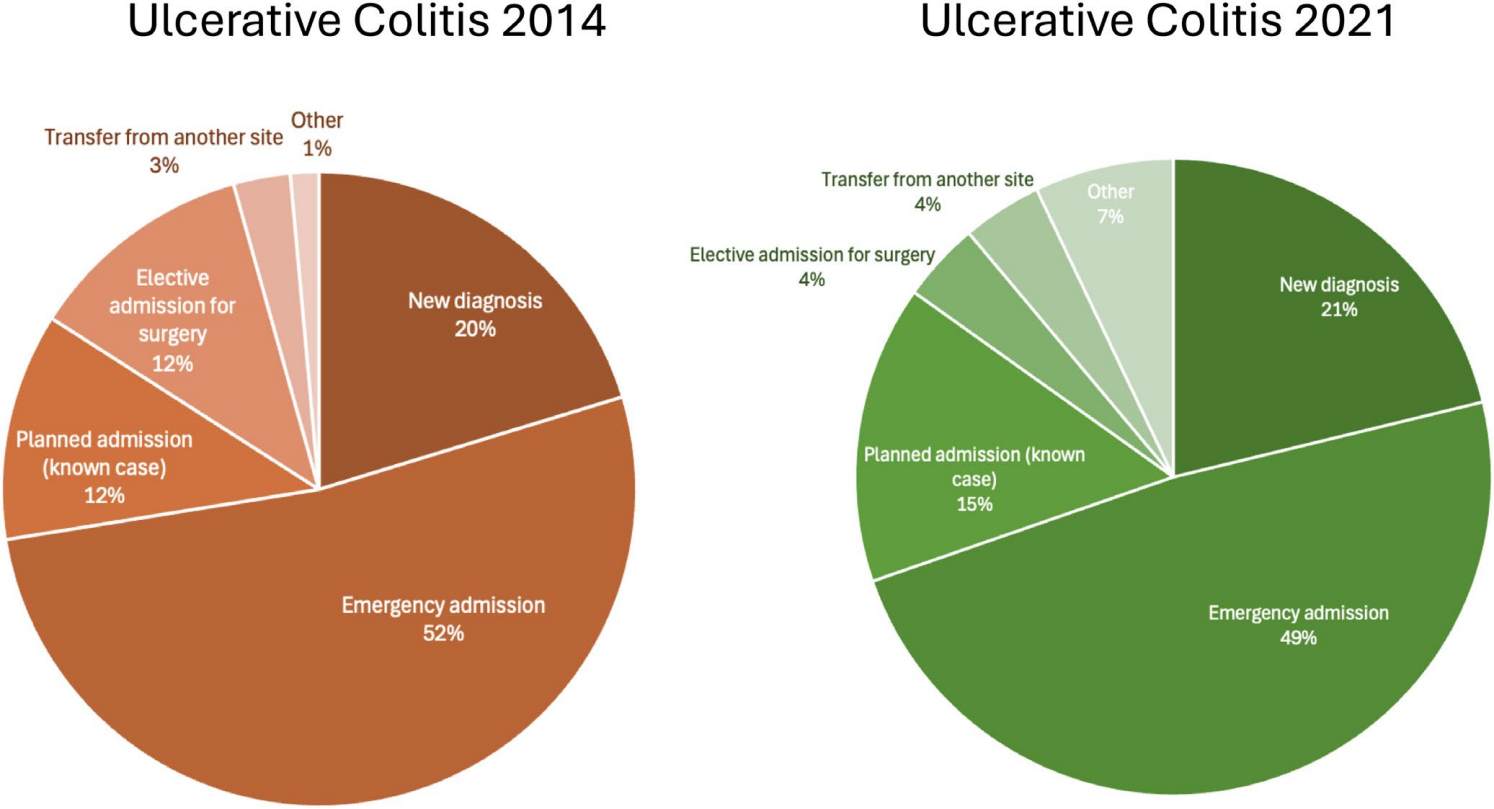
Primary reason for Ulcerative Colitis admissions, expressed as % of total UC admissions in CCA audit.

Emergency admissions made up the majority of reasons for admissions for both 2014 (45%) and 2021 (41%) in CD, as well as UC, with 52% in 2014 and 48% in 2021 (see Figures 4 and 5).

Re‐admissions in the last 30 days for CD remained the same in both years; there was a slight numerical increase in this number for UC‐related re‐admissions (from 23% to 33%, *p* = ns). It should be noted here that 65% of UC patients and 62% of CD patients admitted had active disease at the last clinic review.

There was also a trend towards shorter admission duration for both UC and CD compared to 2014, but this was not statistically significant. In 2014, the mean length of stay for CD with removal of extreme outliers was 6.70 days; this reduced to 5.81 days in 2021 (interquartile range 6.00, median 4.00 days). When including outliers of 213 and 278 days, the mean length of stay was 11.3 days. For UC, there was a reduction in the mean number of days admitted from 10.20 days in 2014 to 4.46 days (interquartile range 4.00, median 3.00 days) in 2021, with one extreme outlier removed. When including one extreme outlier of 280 days, the mean length of stay for UC in 2021 was 5.37 days (see Table 1).

**TABLE 1:** Length of admission from 2014 to 2021.

	UC 2014	UC 2021	CD 2014	CD 2021
Mean length of stay (LOS)^a^	10.20 days	4.46 days	6.70 days	5.81 days
Median LOS	NA	3.00 days	NA	4.00 days
Interquartile range	NA	4.00 days	NA	6.00 days

*Note:* These figures were not statistically significant.

^a^ These figures are with the removal of one extreme outlier in 2021. The mean LOS for UC in 2021 with one extreme outlier (280 days) included was 5.37 days. The mean LOS for CD with the removal of extreme outliers (213 and 278 days) was 11.3 days.

The 2021 audit showed most patients had CD for < 1 year (68%), compared to 2014, where a higher proportion of patients had disease for 1–5 years (33%). The number of patients who had CD for 5–10 years dropped from 24% in 2014 to 3% in 2021 (*p* < 0.01). For UC, there was a higher number of children with disease < 1 years, but this was not statistically significant. There was a reduction in children who had disease 5–10 years, from 18% in 2014 to 4% in 2021 (*p* < 0.05) (See Table 2).

**TABLE 2:** Disease duration compared from 2014 to 2021.

	UC 2014	UC 2021	CD 2014	CD 2021
< 1 year	37%	55%	15%	68%
1–5 years	44%	41%	57%	27%
5–10 years	18%	4%*	24%	3%**
≥ 10 years	NA	NA	3%	3%

*Note:* *p < 0.05. **p < 0.01. This is expressed as a percentage of total CD or UC patient admissions in the audit data.

Anaemia was present in almost half of patients (46% in CD and 45% in UC), similar to the previous 2014 audit (48% in CD and 42% for UC); however, these results were not statistically significant. Malnutrition was present on admission for 37% of CD patients in 2021 (similar to 35% recorded in 2014), and 16% of UC patients in 2021 (lower than 25% recorded in 2014); however, these results were also not statistically significant.

There was a statistically significant reduction in mental health support for CD patients, with psychology review reducing from 50% in 2014 to 20% in 2021 (*p* < 0.05), and psychiatry review from 15% in 2014 to 3% in 2021 (*p* < 0.05). There were similar numerical reductions for UC, but these were not statistically significant. It was noted that almost a quarter of patients for both UC and CD reported psychological comorbidity (in 2021, UC 26% and 30% in CD).

Flexible sigmoidoscopy was carried out in 22% of patients who presented with UC within the first 24 h; this increased to 32% within the first 72 h. Just under half of patients for UC and CD had investigation for *Clostridium difficile* within the first 48 h of admission. Fewer patients had an abdominal x‐ray as part of work up for UC admission, from 32% in 2014 to 17% in 2021 (*p* < 0.05).

**FIGURE 6:**
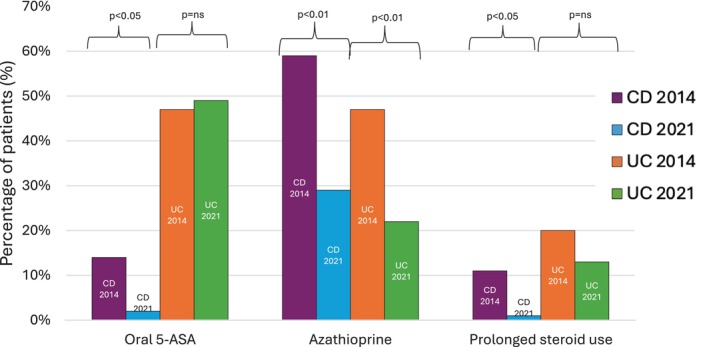
Medication use expressed as the percentage of patients within each UC and CD cohort in 2014 and 2021.

There was a decrease in use of oral 5‐ASA in CD from 14% to 2% (*p* < 0.05). There was also a statistically significant reduction in use of azathioprine in both UC and CD inpatients. For CD, azathioprine use halved from 59% to 29% (*p* < 0.01) for UC, from 47% to 22% (*p* < 0.01). There was a reduction in use of prolonged systemic steroids (> 3 months in the year prior to admission) for CD from 11% to 1% (*p* < 0.05). There was a reduction noted for UC, but it was not statistically significant. See Figure 6 for further detail.

There was a reduction in the use of deep venous thrombosis/pulmonary embolism prophylaxis in both UC and CD. For CD, this audit showed a reduction in use from 19% in 2014 to 8% in 2021 (*p* < 0.05), and for UC, from 30% in 2020 to 10% in 2021 (*p* < 0.01). No thrombotic episodes were recorded.

There was a decrease in requirement for surgery during admission for UC from 16% in 2014 to 5% in 2021 (*p* < 0.05). There was no change in surgery during admission for CD.

**FIGURE 7:**
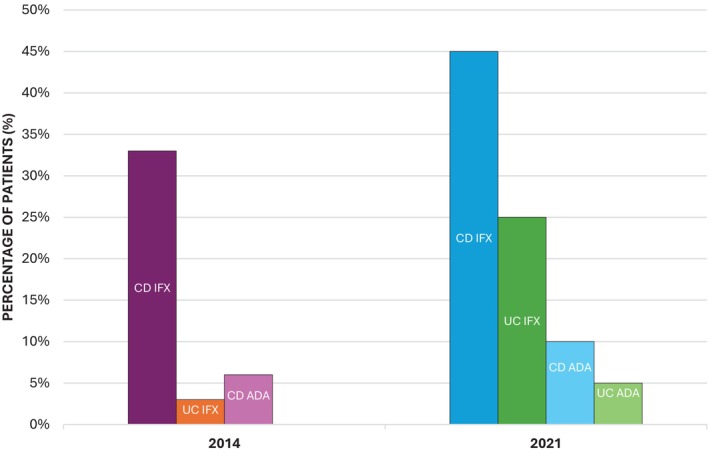
Use of biological therapies expressed as a percentage of patients within each UC and CD subgroup in 2014 and 2021. IFX = infliximab, ADA = adalimumab. Note there was no data available in 2014 for use of adalimumab in ulcerative colitis as not available via PBS.

There was an increase in the number of patients on biological therapies in both CD and UC and included both infliximab and adalimumab. Most patients on infliximab remained on immunomodulators (77.5%), and 50% of patients on adalimumab remained on immunomodulators. We did not have data on UC patients on adalimumab (as it was not available on the Pharmaceutical Benefits Scheme [PBS] for this indication in 2014), and IBD‐unspecified (IBD‐U) patients on infliximab and adalimumab in 2014 so we cannot make comparisons with this data (see Figure 7). There were 56 patients on off‐label medications in this study.

This study captured 24 overnight surgical admissions (Note—this is almost all of the 26 recorded nationally in public hospitals). CD‐related surgery remained stable from 16% in 2014 to 14% in 2021 (*p* = ns); however, for UC, we saw a statistically significant reduction in surgery required from 16% in 2014 to 5% in 2021 (*p* < 0.05). There were more sites that had paediatric surgeons performing ileo‐anal pouch surgery in 2021, with most performed by paediatric surgeons in conjunction with an adult colorectal surgeon (see Table 3).

**TABLE 3:** Surgical procedures performed captured in audit data.

	UC 2014	UC 2021	CD 2014	CD 2021
Small bowel surgery	0	0	2	0
Large bowel surgery	10	6	3 (all hemicolectomies +/− anastomosis or stoma formation)	5 (all hemicolectomies with anastomosis)
Anorectal examination	NA	2	NA	4
Perianal surgery (insertion or removal of anal seton)	NA	1	NA	5
Closure of stoma	NA	1	NA	0
Total	10	10	5	14

*Note:* These are numbers of patient admissions requiring surgery.

## 3 Discussion

This national audit demonstrated the overall change in hospital care for children with IBD in Australia from 2014 to 2021. Over 90% of all IBD admissions for children we captured in the 2021 data, and a detailed audit of 25% of these admissions has allowed a thorough snapshot of the care of children with IBD in hospitals across Australia in this period. While not a complete audit, the non‐selective method of patient auditing (chronological), as well as the large overall percentage of patients captured, gives confidence that this is a representative group.

We noted an ongoing increased incidence of PIBD admissions in the national data, which is consistent with more recent national data from the recent Australian State of the Nation Report, and other global datasets [3, 13, 14, 15] It should be noted that the follow up audit was completed in the midst of the COVID‐19 pandemic. It could be hypothesised that this led to a delay in hospital presentation, particularly for UC which, due to the nature of the symptoms, makes it difficult to avoid the need for acute care. There was also limited capacity for face‐to‐face outpatient reviews, and patients may have been required to present to hospital for escalation in treatment in a timely manner. Escalation of therapy in UC often requires the hospital admission for intravenous steroids, whereas CD escalation in therapy can be completed in the outpatient setting (unless perianal disease), which could explain the increase in UC admissions compared to CD admissions. While this work was not designed to assess the incidence of IBD in Australia, the increase in UC admissions could be related to the increased numbers of patients.

There was a high number of readmission rates for both UC and CD, comparable to international data [9, 10]. With the significant increase in UC admissions, it is noted that 65% of patients had active disease at the last clinic review, suggesting that we need better methods for escalating in therapy in the outpatient setting. Of note, access to IBD nurses had not improved across this timeframe, which has been shown to reduce emergency presentations [16, 17].

In the clinical audit, we saw an apparent increase in CD admissions in the younger age group, which, if representative of the national data, could be explained by increased use of biologics in older children meaning more stable disease in this age group and fewer admissions required in this proportion of patients. Alternatively, or in addition, this may suggest paediatric CD is presenting at an earlier age, or more severely in this group, although it is beyond the scope of this study to comment on overall incidence or prevalence data. Given the younger age at presentation, it is not surprising that many patients require admission at diagnosis for work up.

Despite psychological comorbidity reported in almost a quarter of patients, there was a reduction in mental health support, perhaps reflecting the huge demand in this sector generally and therefore the inability to provide specialist support, especially during the Covid‐19 pandemic [18]. While the pandemic undoubtedly compounded psychological issues, there was no dedicated psychological support for IBD patients in any centre prior to the pandemic, during it or immediately afterwards. Psychological comorbidity leads to poorer patient outcomes, with reduced health‐related quality of life, for patients and carers contributing to the burden of disease [19, 20, 21, 22, 23]. Given this trend it is important to consider alternatives to individual mental health support delivered by psychologists with studies implementing virtual pre‐recorded and tailored programs needed.

Current clinical guidelines for the management of acute severe ulcerative colitis (ASC) recommend early abdominal x‐ray, stool infective screen including *Clostridium difficile* screen, use of DVT/PE prophylaxis and investigation for CMV colitis in patients not responding to early intravenous corticosteroid therapy [24] This study showed that only a third of patients had a flexible sigmoidoscopy at 72 h, just under half of patients had an investigation for *C.difficile*, and only 17% had an abdominal x‐ray as part of the workup. This could be explained by active disease on presentation but not meeting criteria for ASC. This could also explain the reduction in the use of DVT/PE prophylaxis and surgery. It could be a reflection on the increasing confidence of the medical teams on their ability to avoid surgery and/or prolonged admissions with earlier use of advanced medical therapies, especially anti‐TNF medications. It is beyond the scope of this study to determine why international guidelines were not followed but this is an important area for future work including exploring factors influencing adherence to existing guidelines, identifying barriers in implementation and determining whether updates are required at a local or international level are warranted.

The reduction in use of 5‐ASA therapy in Crohn's disease is an expected finding from 2014 to 2021 with better understanding of therapy options [25].

There was a reduction in use of azathioprine in both UC and CD, with no related increase in use of mercaptopurine or methotrexate suggesting more patients on monotherapy for their anti‐TNF. This is presumably due to safety concerns [26, 27, 28] but may also reflect again the increasing use of anti‐TNF therapy including its use as monotherapy [29].

Positively, despite an increase in UC admissions, it is suggested that there is a better use of rescue medications, reflected by the trend in shorter admissions, reduction in the use of steroids for longer than 3 months in the last year, fewer patients requiring surgery, and with disease for 5–10 years requiring admissions.

Overall, this study demonstrates the changing pattern of IBD hospital care in Australia over the last decade. While the proportion of UC patients has increased, with of Crohn's disease have remained stable. Unfortunately, it appears that despite active disease in an outpatient setting, there has not been an improvement in preventing emergency admissions, perhaps due to that lack of IBD nurse availability compounded by the COVID‐19 pandemic. Many other parameters have changed in line with international patterns of medication use, with a pleasingly low rate of surgical intervention. It is worth mentioning the off‐label medication use, as this may have prevented the need for further surgical intervention. Mental health remains a significant unmet need in young people with IBD. This work provides useful data for policymakers and advocates in prioritising the needs of young people with IBD in Australia and elsewhere.

## Author Contributions

All authors have made substantial contributions to all of the following: (1) the conception and design of the study, or acquisition of data, or analysis and interpretation of data, (2) drafting the article or revising it critically for important intellectual content, (3) final approval of the version to be submitted.

## Funding

This work was supported by the Department of Health and Aged Care, Australian Government.

## Conflicts of Interest

The authors declare no conflicts of interest.

## Supporting information


**Data S1:** Ulcerative colitis paediatric clinical audit.

## Data Availability

The data that support the findings of this study are available from the corresponding author upon reasonable request.
