# Supplementary material for: Changes in Hospital Care for Children With IBD Across Australia From 2014 to 2022
Source: J Paediatr Child Health. 2026 Jun 1;62(7):1235–43. doi: 10.1111/jpc.70424 (PMC13378197; doi:10.1111/jpc.70424)
Supplement: Supplementary file 1 — Data S1: Ulcerative colitis paediatric clinical audit. [file JPC-62-1235-s001.docx]

**Ulcerative Colitis Paediatric Clinical Audit**

**Admission/Mortality**

**You will need to complete the first page 'Admission/mortality' prior to commencing subsequent pages of the audit**

**Patient demographics/diagnosis**

1.1. Given name

1.2. Surname

1.3. What is the patient's date of birth? DD/MM/YYYY

a. Admission age (calculated)

1.4. What is the patient's UR (hospital record) number?

1.5. Gender  Male  Female  Other

**Admission**

1.6. What was the date of admission? DD/MM/YYYY

1.7. What was the primary reason for admission?

 New diagnosis of UC
 Emergency admission for active UC
 Planned admission for active UC (known case)
 Elective admission for surgery
 Transfer from another site - for IBD care
 Not IBD-Related
 Other

a. If 'Other' please specify

1.8 What was the source of admission? (select all that apply)

 ED admission
 Referred by GP
 Advised to attend via IBD nurse helpline
 Referred in from Hospital OPD
 Referred in from GE specialist rooms
 Referred in from surgical specialist rooms
 Transfer from another site
 Other

a. If 'Other' please specify

1.9. Has the patient had previous overnight admissions with UC in the two years prior to this admission at this hospital?  Yes  No

a. If yes, how many times in the two years prior to this admission?

b. Has there been a UC related admission within the last 30 days? Yes No

c. Has this patient already been included in this audit?

 Yes  No

i. If yes, what is the patient audit number for the last audited admission? (generated by the web tool)

**Discharge/Mortality**

1.10. The patient was:

 Discharged home
 Discharged at own risk
 Discharged to nursing home or rehabilitation centre
 Transferred to another centre for surgery
 Transferred to another centre for medical management
 Deceased

a. What was the date of discharge? DD/MM/YYYY

b. What was the date of death? DD/MM/YYYY

c. Was the death UC-related?  Yes  No  Not applicable (N/A)

d. Please enter further details of death with specific reference to post-operative complications, infections, adverse events related to medications, thromboembolic disease, malignancy and other causes:

**Extent and Severity of UC**

**Initial assessment during the first full day following admission**

2.1. Was duration of disease stated in admission notes?  Yes  No

2.2. What was the month/year of diagnosis? MM/YYYY Month not known

2.3. Was the extent of colitis at the most recent assessment recorded in the admission notes?  Yes  No

a. If yes, was the disease: (Select all that apply)

 Ulcerative proctitis (E1)
 Left-sided UC (distal to splenic flexure) (E2)
 Extensive (hepatic flexure distally) (E3)
 Pancolitis (proximal to hepatic flexure (E3)
 Never severe* (S0)
 Ever severe* (S1)
 IBD-U
 Unknown

*Severe defined by Pediatric Ulcerative Colitis Activity Index (PUCAI)

2.4. Was there documentation of the severity of disease activity recorded in the first 24 hours?
E.g. PUCAI?
Criteria used for the assessment of acute severe UC include the number of bloody stools per day, and the presence of fevers, tachycardia, anaemia, or an elevated ESR (or CRP)

 Yes  No

2.5 Were the following items documented in the clinical record?

a. Number of loose stools passed in the first full day following admission? Yes No N/A

 Patient had stoma

b. Number of bloody stools passed in the first full day following admission Yes No N/A

2.6 Did the notes record the current presence of any of the following?

a. Fevers Yes No Not documented

b. Presence of nocturnal stools Yes No Not documented

c. Presence of urgency or incontinence Yes No Not documented

d. Mouth ulcers Yes No Not documented

e. Arthralgia Yes No Not documented

f. Arthritis Yes No Not documented

g. Ankylosing spondylitis  Yes  No  Not documented

h. Erythema nodosum  Yes  No  Not documented

i. Pyoderma gangrenosum  Yes  No  Not documented

j. Iritis  Yes  No  Not documented

k. Anal fissure  Yes  No  Not documented

l. Fistula  Yes  No  Not documented

m. Abscess  Yes  No  Not documented

n. Malnutrition  Yes  No  Not documented

**Comorbidity**

2.7. Were any significant comorbid diseases/conditions documented? (select all that apply)

 Yes  None recorded  Statement that patient had no relevant comorbidities

a. Which comorbidities were documented?

 Cardiovascular
 Respiratory
 Renal
 Diabetes
 Liver disease
 Active cancer
 Psychological condition
 Other

i. Specify details of comorbid diseases

**Medication on admission**

2.8. Was the patient taking treatment for ulcerative colitis on admission? Yes No Not stated

a. What treatments was the patient taking? (Select all that apply)

 Sulfasalazine
 Oral 5-ASA
 Topical 5-ASA
 Oral corticosteroids
 Topical corticosteroids
 Mercaptopurine
 Azathioprine
 Methotrexate
 Antibiotics
 Dietary therapy
 Allopurinol
 Anti-TNF agent
 Other (e.g. trial medication or Complementary medicine)

i. If 'Other' please specify

b. Was there an estimate of compliance recorded?  Yes  No

**Smoking status**

2.9. What was the smoking status of the patient?

 Current smoker
 Not currently smoking
 Not documented

**Other assessment during admission**

**Prolonged steroid use**

2.10. In the 12 months prior to admission was the patient taking oral steroids for UC (at any time) for >3 months?  Yes  No  Unknown

a. Was an appropriate dose reduction planned?  Yes  No  Unknown

b. Was bone protection used?  Yes  No  Unknown

c. Had a DEXA scan been done within 5 years?  Yes  No  Unknown

d. If yes (>3 m steroids), what steroid sparing strategies were tried? (Select all that apply)

 Thiopurine
Methotrexate
Anti-TNF agent
 None
Other

i. If 'Other' please specify

e. What was the outcome of the steroid-sparing strategy?

 Ongoing steroid-sparing therapy
 Stopped due to intolerance
 Stopped due to lack of clinical benefit
 Successful steroid cessation
 Other

1. If 'Other' please specify

**Weight assessment and dietetic support during admission**

2.11. Was a dietetic assessment recorded? Yes  No

2.12. Was a formal nutritional risk assessment documented in the patient record? (e.g. PSGNA/PNST, STAMP, STRONGkids or PYMS  Yes  No

a. By whom?  Nurse  Doctor  Dietitian  Nutrition assistant  Unclear

2.13. Was the patient’s weight recorded within two days of admission?  Yes  No

a. Was the patient’s height recorded?  Yes  No

2.14. Was the patient’s weight recorded within two days of discharge?  Yes  No

2.15. Was BMI recorded?  Yes  No

2.16. Was it documented that a dietitian saw the patient?  Yes  No  N/A (thought to be well nourished/ not needed)

2.17. Was dietary treatment recommended?  Yes  No  Not recorded

**Investigation**

2.18 What were the admission results (within 24 hours) for the following tests?

a. CRP (mg/L)  Not documented

b. Hct (%)  Not documented

c. Hb (g/dL)  Not documented

d. Albumin (g/L)  Not documented

e. Faecal calprotectin (µg/g)  Not documented

2.19. Was a stool sample sent for stool culture/PCR within 48 hours of admission? Yes No  N/A

a. Was it positive? Yes No

2.20. Was a stool sample sent for Clostridium difficile toxin within 48 hours of admission? Yes No N/A

a. Was it positive? Yes No

2.21. Was flexible sigmoidoscopy or colonoscopy carried out within 24 hours of admission in patients presenting with acute severe UC? Yes No N/A

2.22. Was flexible sigmoidoscopy or colonoscopy carried out between 24 to 72 hours of admission in patients presenting with acute severe UC?  Yes  No  N/A

2.23. Were biopsies taken for histology?  Yes  No

a. For CMV?  Yes  No

2.24 What imaging was used during the admission? (select all that apply)

 No imaging performed
 AXR
 Abdominal ultrasound
 Abdominal CT scan
 Other

i. If 'Other' please specify

**Care Team**

**IBD team/ward (who looked after them?)**

3.1. Which specialty was responsible for the patient's care 24 hours after admission?

 Paediatric acute or general medicine
 Acute or general medicine
 Paediatric general surgery
 General surgery
 Paediatric gastroenterology
 Gastroenterology
 Paediatric colorectal surgery
 Colorectal surgery
 Other

a. If 'Other' please specify

3.2. Was a paediatric gastroenterology consultant or paediatric registrar/fellow consulted? Yes No Not required  Not documented

3.3 Was a paediatric surgeon, paediatric colorectal surgeon, colorectal surgeon or respective registrars consulted? Yes  No  Not required  Not documented

3.4. Is there documentation that an IBD nurse specialist saw the patient during admission? Yes  No

3.5. Was the patient cared for on a specialist gastroenterology ward?  Yes  No

a. Which type of ward?

 Adult medical/gastroenterology
 Adult surgical
 Paediatric medical
 Paediatric gastroenterology
 Joint paediatric gastroenterology/surgical
 Other

3.6. While admitted, did the patient receive any short term psychological support?  Yes  No

a. Who provided the short term psychological support?

 Psychologist
 Psychiatrist
 Social worker
 Pastoral care
 Other

i. Please specify

3.7. While on ward, did the patient receive short-term psychotropic medication (e.g., anxiolytic) to help with adjustment issues (e.g., sleeping difficulties, anxiety)?  Yes  No  NA

**Medical intervention**

**If the patient was admitted electively for surgery, ignore the medical intervention section other than 4.1.**

**Use of anti-thrombotic therapy**

4.1. Was the patient given DVT/PE prophylaxis?  Yes  No  Contraindicated

a. If contraindicated, why?

4.2. Did the patient have a thrombotic episode during this admission?  Yes  No

a. What type of episode was it?  DVT  PE  Other

i. If 'Other' please specify

**Other medical therapy**

4.3. Were corticosteroids initiated during this admission? Yes  No

a. If 'yes', what was the route of administration?

 IV corticosteroids
 Oral corticosteroids
 Topical corticosteroids

4.4 Which other therapies were started during the admission? (select all that apply)

 None
 5-Aminosalicylates
 Thiopurine therapy
 Methotrexate
 Cyclosporin
 Anti-TNF
 Other nutrition support
 Other

i. If 'Other' please specify

4.5. Is there documentation of the patient having been discussed at a multidisciplinary team meeting?  Yes  No

**Surgical Intervention**

**Surgical therapy**

5.1. Did the patient have surgery on this admission?  Yes  No

a. What was the date of surgery? DD/MM/YYYY

b. Was there a delay of more than 48 hours between decision to operate and surgery?

 Yes  No  Unclear

i. What was the reason for the delay?

 Improvement in severity of UC
 Cancelled due to lack of theatre time or other resource-related reasons
 Cancelled for clinical reasons (e.g to correct hyperkalaemia)
 Patient declined surgery or needed time to consider
 Unclear
 Other

i. If 'Other' please specify

5.2. Was the ASA status recorded on an anaesthetic chart? Yes No

a. What was the status? 1 2 3 4 5 N/A

5.3 What were the indications for this surgery? (select all that apply)

 Failure of medical therapy
 Toxic megacolon
 Perforation
 Abscess
 Bleeding
 Obstruction
 Dysplasia
 Cancer
 Formation of ileostomy
 Closure of stoma
 Completion proctectomy
 Other indication

1. If 'Other' please specify

5.4 Type of intervention (select all that apply)

 Proctocolectomy
 Subtotal colectomy
 Completion proctectomy
 Formation of ileal pouch-anal anastomosis
 Formation of ileostomy
 Revision of stoma
 Closure of stoma
 Drainage of abscess
 Division of adhesions
 Perineal procedure
 Other intervention

1. If 'Other' please specify

5.5. Was the surgery done laparoscopically/ laparoscopically assisted? Yes No Unclear

5.6. Was the patient seen by a stomal therapy nurse during this admission? Yes No Unclear

5.7. Was the patient seen by a stomal therapy nurse prior to surgery? Yes No Unclear

**Surgical complications**

5.8. Did the patient develop postoperative complications? (Select all that apply) Yes No

 Wound infection
 Rectal stump complications
 Intra-abdominal bleeding
 Intra-abdominal abscess
Anastomotic leakage
 Stoma complications
 Deep vein thrombosis (DVT)
 Pulmonary embolus (PE)
 Small bowel obstruction
 Ileus
 Total parenteral nutrition (TPN)
 Cardiac
 Respiratory
 Clostridium difficile-associated diarrhoea (CDAD)
 Malnutrition
 Reoperation (for any reason)

i. Please specify reoperation reason

 Other

ii. Please specify 'other' complication details

**Anaemia**

5.9. Was the patient anaemic on admission?  Yes  No  Not recorded

a. Was the anaemia noted or commented on by the treating team?  Yes  No

5.10. Was anaemia (at presentation or during hospitalization) due to iron deficiency?

 Yes  No  Other cause or uncertain  Not recorded

a. What treatment was administered? (Select all that apply)

 Oral iron
 IV iron
 Blood transfusion
 Nutritional advice
Not recorded

**Discharge Arrangements**

**This section is only required if you answered that the patient was 'discharged home' or 'at own risk' earlier in the survey**

6.1. Was the patient taking oral steroids on discharge? Yes No N/A

a. Was a steroid reduction program stated on discharge?  Yes  No  N/A

6.2. Were bone protection agents prescribed?  Yes  No  N/A

6.3. Was ongoing nutritional supplementation recommended on discharge?  Yes  No  N/A

6.4. Were arrangements made for follow-up by a dietitian?  Yes  No  N/A

6.5. Was the patient on immunosuppressives on discharge?  Yes  No  N/A

a. Was a plan for safety monitoring implemented?  Yes  No  N/A

6.6. Was there a plan for maintenance anti-TNF on discharge?  Yes  No  N/A

a. Was a plan for safety monitoring implemented?  Yes  No  N/A

6.7. Were psychological/behavioural factors identified to contribute to poor disease management (e.g., significant anxiety/depression leading to non-adherence)

 Yes  No  Unclear

a. If yes, was an outpatient plan put in place to help the patient address this? Yes  No  Unclear

6.8. Was the plan for follow-up documented in the discharge summary?  Yes No

6.9. Was the discharge summary sent/faxed/emailed to the patient’s general practitioner?

 Yes  No  Unclear

**Outpatient Care Prior to Admission**

7.1. Did the patient have previous outpatient visits or private practice consultation for IBD?

 Yes  No  Unknown

If yes:

a. How many times was the patient seen in the 12 months prior to the start date of this admission?  Unknown

b. Was disease active at last OPD appointment or private practice review?

 Yes No Unknown

If no,

a.           Was the patient previously referred to outpatients for diagnostic review:

 Within the last four weeks

 More than four weeks ago

 Not referred into outpatients

**Crohn’s Disease Paediatric Clinical Audit**

**Admission / Mortality**

**You will need to complete the first page 'Admission/mortality' prior to commencing subsequent pages of the audit**

**Patient demographics/diagnosis**

1.1. Given name

1.2. Surname

1.3. What is the patients' date of birth? DD/MM/YYYY

a. Admission age (calculated)

1.4. What is the patient's UR (hospital record) number?

1.5. Gender  Male  Female  Other

**Admission**

1.6. What was the date of admission? DD/MM/YYYY

1.7. What was the primary reason for admission?

 New diagnosis of Crohn's disease
 Emergency admission for active Crohn's disease
 Planned admission for Crohn's disease (known case)
 Elective admission for surgery
 Transfer from another site - for IBD care
 Not IBD-Related
 Other

a. If 'Other' please specify

1.8 What was the source of admission? (select all that apply)

 ED admission
 Referred by GP
 Advised to attend via IBD nurse helpline
 Referred in from Hospital OPD
 Referred in from GE specialist rooms
 Referred in from surgical specialist rooms
ð Transfer from another site
 Other

a. If 'Other' please specify

1.9. Has the patient had previous overnight admissions with CD in the two years prior to this admission at this hospital?  Yes  No

a. If yes, how many times in the two years prior to this admission?

b. Has there been a CD-related admission within the last 30 days?  Yes  No

c. Has this patient already been included in this audit?
  Yes  No

i. If yes, what is the patient audit number for the last audited admission? (generated by the web tool)

**Discharge/Mortality**

1.10. The patient was:

 Discharged home
 Discharged at own risk
 Discharged to nursing home or rehabilitation centre
 Transferred to another centre for surgery
 Transferred to another centre for medical management
 Deceased

a. What was the date of discharge? DD/MM/YYYY

b. What was the date of death? DD/MM/YYYY

c. Was the death CD-related?  Yes  No  Not applicable (N/A)

d. Please enter further details of death with specific reference to post-operative complications, infections, adverse events related to medications, thromboembolic disease, malignancy and other causes:

**Extent and Severity of CD**

**Initial assessment during the first full day following admission**

2.1. Was duration of disease stated in admission notes?  Yes  No

2.2. What was the month/year of diagnosis? MM/YYYY  Month not known

2.3. Was the extent of Crohn's disease at the most recent assessment recorded in the admission notes?  Yes  No

a. If yes, was the disease: (Select all that apply)

 Distal 1/3 ileum ± limited cecal disease (L1)
 Colonic (L2)
 Ileo-colonic (L3)
 Upper disease proximal to Ligament of Treitz (L4a)
 Upper disease distal to Ligament of Treitz proximal to distal 1/3 ileum (L4b)
 Unknown

2.4 Were the following items documented in the clinical record?

a. Number of liquid stools per day  Yes  No  N/A Patient had stoma

b. The presence of blood in the stools  Yes  No  N/A

c. General wellbeing  Yes  No  N/A

d. Perineal examination  Yes  No  N/A

2.5 Did the notes record the current presence of any of the following?

a. Fevers  Yes  No  Not documented

b. Active perineal disease  Yes  No  Not documented

c. Abdominal mass  Yes  No  Not documented

d. Abdominal pain  Yes  No  Not documented

e. Mouth ulcers  Yes  No  Not documented

f. Oro-facial granulomatosis  Yes  No  Not documented

g. Arthralgia  Yes  No  Not documented

h. Arthritis  Yes  No  Not documented

i. Ankylosing spondylitis  Yes  No  Not documented

j. Erythema nodosum  Yes  No  Not documented

k. Pyoderma gangrenosum  Yes  No  Not documented

l. Iritis  Yes  No  Not documented

m. Anal fissure  Yes  No  Not documented

n. Fistula  Yes  No  Not documented

o. Abscess  Yes  No  Not documented

p. Malnutrition  Yes  No  Not documented

2.6. Was a paediatric Crohn's disease activity index (PCDAI) score recorded?  Yes  No

**Comorbidity**

2.7. Were any significant comorbid diseases/conditions documented? (select all that apply)

 Yes  None recorded  Statement that patient had no relevant comorbidities

1. Which comorbidities were documented?

 Cardiovascular
 Respiratory
 Renal
 Diabetes
 Liver disease
 Active cancer
 Psychological condition
 Other

i. Specify details of comorbid diseases

**Medication on admission**

2.8. Was the patient taking treatment for Crohn's disease on admission?  Yes  No  Not stated

a. What treatments was the patient taking? (select all that apply)

 Sulfasalazine
 Oral 5-ASA
 Topical 5-ASA
 Oral corticosteroids
 Topical corticosteroids
 Mercaptopurine
 Azathioprine
 Methotrexate
ð Antibiotics
 Exclusive Enteral Nutrition
 Other Dietary therapy

 Allopurinol  Anti-TNF agent
 Other (e.g. trial medication or complementary medicine)

i. If 'Other' please specify

b. Was there an estimate of compliance recorded?  Yes  No

**Smoking status**

2.9. What was the smoking status of the patient?

 Current smoker
 Not currently smoking
 Not documented

**Other assessment during admission**

**Prolonged steroid use**

2.10. In the 12 months prior to admission was the patient taking oral steroids for CD (at any time) for >3 months?  Yes  No  Unknown

a. Was an appropriate dose reduction planned?  Yes  No  Unknown

b. Was bone protection used?  Yes  No  Unknown

c. Had a DEXA scan been done within 5 years?  Yes  No  Unknown

d. If yes (>3 m steroids), what steroid sparing strategies were tried? (Select all that apply)

 Thiopurine
 Methotrexate
 Anti-TNF agent
 None
 Other

i. If 'Other' please specify

e. What was the outcome of the steroid-sparing strategy?

 Ongoing steroid-sparing therapy
 Stopped due to intolerance
 Stopped due to lack of clinical benefit
 Successful steroid cessation
 Other

1. If 'Other' please specify

**Weight assessment and dietetic support during admission**

2.11. Was a dietetic assessment recorded?  Yes  No

2.12. Was a formal nutritional risk assessment documented in the patient record? (e.g. PSGNA/PNST, STAMP, STRONGkids or PYMS)  Yes  No

a. By whom?  Nurse  Doctor  Dietitian  Nutrition assistant  Unclear

2.13. Was the patient’s weight recorded within two days of admission?  Yes  No

a. Was the patient’s height recorded?  Yes  No

2.14. Was the patient’s weight recorded within two days of discharge?  Yes  No

2.15. Was BMI recorded?  Yes  No

2.16. Was it documented that a dietitian saw the patient?  Yes  No N/A (thought to be well nourished/not needed)

2.17. Was dietary treatment recommended? Yes  No  Not recorded

**Investigations**

2.18 What were the admission results (within 24 hours) for the following tests

a. CRP (mg/L)  Not documented

b. Hb (g/dL)  Not documented

c. Albumin (g/L)  Not documented

d. Faecal calprotectin (µg/g)  Not documented

e. Haematocrit (%)  Not documented

f. Esr (mm/hr)  Not documented

2.19. Was a stool sample sent for stool culture/PCR within 48 hours of admission?  Yes  No N/A

a. Was it positive?  Yes  No

2.20. Was a stool sample sent for *Clostridium difficile* toxin within 48 hours of admission?
 Yes  No  N/A

a. Was it positive?  Yes  No

2.21 What imaging was used during the admission? (select all that apply)

ð No imaging performed
 AXR
 Abdominal ultrasound (not small bowel specific)
 Specific small bowel ultrasound
 Abdominal CT scan
 MR Enterography
 Other

1. If 'Other' please specify

**Care Team**

**IBD team/ward (who looked after them)**

3.1. Which specialty was responsible for the patient's care 24 hours after admission?

 Paediatric acute or general medicine
 Acute or general medicine
 Paediatric general surgery
 General surgery
 Paediatric gastroenterology
 Gastroenterology
 Paediatric colorectal surgery
 Colorectal surgery
 Other

a. Please specify

3.2. Was a paediatric gastroenterology consultant or paediatric registrar/fellow consulted?
 Yes  No  Not required  Not documented

3.3. Was a paediatric surgeon, paediatric colorectal surgeon, colorectal surgeon or respective registrars consulted?
Yes  No  Not required  Not documented

3.4. Is there documentation that an IBD nurse specialist saw the patient during admission?
 Yes  No

3.5. Was the patient cared for on a specialist gastroenterology ward?  Yes  No

a. Which type of ward?

 Adult medical/gastroenterology
 Adult surgical
 Paediatric medical
 Paediatric gastroenterology
 Joint paediatric gastroenterology/surgical
 Other

3.6. While admitted, did the patient receive any short term psychological support?  Yes  No

a. Who provided the short term psychological support?

 Psychologist
 Psychiatrist
 Social worker
 Pastoral care
 Other

i. Please specify

3.7. While on ward, did the patient receive short-term psychotropic medication (e.g., anxiolytic) to help with adjustment issues (e.g., sleeping difficulties, anxiety)?  Yes  No  NA

**Medical Intervention**

**If the patient was admitted electively for surgery, ignore the medical intervention section other than 4.1**

**Use of anti-thrombotic therapy**

4.1. Was the patient given DVT/PE prophylaxis?  Yes  No  Contraindicated

a. If 'contraindicated', why?

4.2. Did the patient have a thrombotic episode during this admission?  Yes  No

a. What type of episode was it?  DVT  PE  Other

i. If 'Other' please specify

**Other medical therapy**

4.3. Were corticosteroids initiated during this admission?  Yes  No

a. If 'yes', what was the route of administration?

 IV corticosteroids
 Oral corticosteroids
 Topical corticosteroids

4.4 Which other therapies were started during the admission? (select all that apply)

 None
 5-Aminosalicylates
ð Thiopurine therapy
 Methotrexate
 Anti-TNF
 Other nutrition support (e.g. EEN, supplemental nutrition , CDED , SCD, PEN)
 Other

i. If 'Other' please specify

4.5. Is there documentation of the patient having been discussed at a multidisciplinary team meeting?  Yes  No

**Surgical Intervention**

**Surgical therapy**

5.1. Did the patient have surgery on this admission?  Yes  No

a. What was the date of surgery? DD/MM/YYYY

b. Was there a delay of more than 48 hours between decision to operate and surgery?
 Yes  No  Unclear

i. What was the reason for the delay?

 Improvement in severity of CD
 Cancelled due to lack of theatre time or resource related reasons
 Cancelled for clinical reasons (e.g to correct hyperkalaemia)
 Patient declined surgery or needed time to consider
 Unclear
 Other

i. If 'Other' please specify

5.2. Was the ASA status recorded on an anaesthetic chart?  Yes  No

a. What was the status?  1  2  3  4  5  N/A

5.3 What were the indications for this surgery? (select all that apply)

 Obstruction
 Perforation
 Abscess
 Formation of ileostomy
 Closure of stoma
 Failure of medical therapy
 Bleeding
 Completion proctectomy
 Dysplasia
 Cancer
ð Fistula
 Other indication

i. If 'Other' please specify

5.4 Type of intervention (select all that apply

 Ileocolonic resection
 Ileal/jejunal resection
 Stricturoplasty
 Segmental/extended colectomy
 Subtotal colectomy
 Proctocolectomy
 Resection of Intra-abdominal fistula
 Completion proctectomy
 Drainage of abscess
 Formation of ileostomy or colostomy
 Revision of stoma
 Closure of stoma
 Perineal procedure
 Division of adhesions
 Seton Insertion
 Other intervention

i. If 'Other' please specify

5.5. Was the surgery done laparoscopically or laparoscopically assisted?  Yes  No  Unclear

5.6. Was the patient seen by a stomal therapy nurse during this admission?  Yes  No  Unclear

5.7. Was the patient seen by a stomal nurse prior to surgery?  Yes  No  Unclear

**Surgical complications**

5.8. Did the patient develop postoperative complications? (Select all that apply)  Yes  No

 Wound infection
 Rectal stump complications
 Intra-abdominal bleeding
 Intra-abdominal abscess
 Anastomotic leakage
 Stoma complications
 Deep vein thrombosis (DVT)
 Pulmonary embolus (PE)
 Small bowel obstruction
 Ileus
 Total parenteral nutrition (TPN)
ð Cardiac
 Respiratory
 *Clostridium difficile*-associated diarrhoea (CDAD)
 Malnutrition
 Reoperation (for any reason)

i. Please specify reoperation reason

 Other

ii. Please specify 'other' complication details

**Prevention of post-operative recurrence**

5.9. Was the patient newly prescribed any of the following drugs on discharge, i.e. additional to those on presentation? (select all that apply)  Yes  No

Azathioprine
Mercaptopurine
 Metronidazole
 5-ASA
 Methotrexate
 Infliximab
 Adalimumab
 Other

i. If 'Other' please specify

**Anaemia**

5.10. Was the patient anaemic on admission?  Yes  No  Not recorded

a. Was the anaemia noted or commented on by the treating team?  Yes  No

5.11. Was anaemia (at presentation or during hospitalization) due to iron deficiency?

 Yes  No Other cause or uncertain Not recorded

a. What treatment was administered? (select all that apply)

 Oral iron
 IV iron
 Blood transfusion
 Nutritional advice
 Not recorded

**Discharge Arrangements**

**This section is only required if you answered that the patient was 'discharged home' or 'at own risk' earlier in the survey**

6.1. Was the patient taking oral steroids on discharge?  Yes  No  N/A

a. Was a steroid reduction program stated on discharge?  Yes  No  N/A

6.2. Were bone protection agents prescribed?  Yes  No  N/A

6.3. Was ongoing nutritional supplementation recommended on discharge?
 Yes  No  N/A

6.4. Were arrangements made for follow-up by a dietitian?  Yes  No  N/A

6.5. Was the patient on immunosuppressives on discharge?  Yes  No  N/A

a. Was a plan for safety monitoring implemented?  Yes  No  N/A

6.6. Was there a plan for maintenance anti-TNF on discharge?  Yes  No  N/A

a. Was a plan for safety monitoring implemented?  Yes  No  N/A

6.7. Were psychological/behavioural factors identified to contribute to poor disease management (e.g., significant anxiety/depression leading to non-adherence)
 Yes  No  unclear

a. If yes, was an outpatient plan put in place to help the patient address this?
 Yes  No  unclear

6.8. Was the plan for follow-up documented in the discharge summary?  Yes  No

6.9. Was the discharge summary sent/faxed/emailed to the patient’s general practitioner?
 Yes  No  N/A

**Outpatient Care Prior to Admission**

7.1. Did the patient have previous outpatient visits or private practice consultation for IBD?
 Yes  No  Unknown

If yes

a. How many times was the patient seen in the 12 months prior to the start date of this admission?  Unknown

b. Was disease active at last OPD appointment or private practice review?
 Yes  No  Unknown

If no,

a.           Was the patient previously referred to outpatients for diagnostic review:

 Within the last four weeks

 More than four weeks ago

 Not referred into outpatients
